# To “Z” or not to “Z”: Z-RNA, self-recognition, and the MDA5 helicase

**DOI:** 10.1371/journal.pgen.1009513

**Published:** 2021-05-13

**Authors:** Alan Herbert

**Affiliations:** Discovery, InsideOutBio, Charlestown, Massachusetts, United States of America; National Cancer Institute, UNITED STATES

## Abstract

Double-stranded RNA (dsRNA) is produced both by virus and host. Its recognition by the melanoma differentiation–associated gene 5 (MDA5) initiates type I interferon responses. How can a host distinguish self-transcripts from nonself to ensure that responses are targeted correctly? Here, I discuss a role for MDA5 helicase in inducing Z-RNA formation by Alu inverted repeat (AIR) elements. These retroelements have highly conserved sequences that favor Z-formation, creating a site for the dsRNA-specific deaminase enzyme ADAR1 to dock. The subsequent editing destabilizes the dsRNA, ending further interaction with MDA5 and terminating innate immune responses directed against self. By enabling self-recognition, Alu retrotransposons, once invaders, now are genetic elements that keep immune responses in check. I also discuss the possible but less characterized roles of the other helicases in modulating innate immune responses, focusing on DExH-box helicase 9 (DHX9) and Mov10 RISC complex RNA helicase (MOV10). DHX9 and MOV10 function differently from MDA5, but still use nucleic acid structure, rather than nucleotide sequence, to define self. Those genetic elements encoding the alternative conformations involved, referred to as flipons, enable helicases to dynamically shape a cell’s repertoire of responses. In the case of MDA5, Alu flipons switch off the dsRNA-dependent responses against self. I suggest a number of genetic systems in which to study interactions between flipons and helicases further.

Recent findings highlight the central role of Z-RNA in regulating innate immune responses. Here, I describe the discovery of Z-DNA and Z-RNA and of the genetic evidence for their involvement in type I interferon responses. I highlight the important part played by the helicase melanoma differentiation–associated gene 5 (MDA5, encoded by *Ifih1*) in modulating the formation of Z-RNA by Alu elements (named for the restriction enzyme that cuts twice within them) to prevent activation of immune responses against host-encoded double-stranded RNAs (dsRNAs). I then describe ways that other helicases, like DExH-box helicase 9 (DHX9) that has a preference for Alu sequences, and Mov10 RISC complex RNA helicase (MOV10) that is also involved in self-recognition, potentially modulate nucleic acid conformation to dynamically revise a cell’s response repertoire as the context changes.

## The background

The discovery of novel nucleic acid structures always raises questions about their biological role. So, it was with left-handed Z-DNA helix named for its zigzag backbone, with its base pairs turned upside down relative to the right-handed B-DNA and A-RNA structures. The atomic detail of Z-DNA was unexpectedly revealed in the first ever DNA crystal [1]. The possibility of a left-handed helix had been anticipated by the earlier observations of Pohl and Jovin [2], who noted an inversion of the optical properties of the right-handed (dG-dC)_n_ DNA polymer when it was placed in 3.5M salt. Once the Z-DNA structure was detailed, work in the Wang lab showed that the higher energy Z-DNA conformer could form from B-DNA without strand cleavage under conditions of topological stress generated by an RNA polymerase unwinding the DNA helix during the act of synthesizing a transcript [3]. Formation of Z-DNA relieved the stress 5′ to the polymerase faster than a topoisomerase could relax it [4]. Subsequent measurements demonstrated that the energetic cost to form the Z-helix is lowest for alternating guanine, cytosine repeats, but the amount varies with base modification [5]. The work lead to the concept of flipons, which are genomically encoded sequences capable of flipping under physiological condition from a lower energy right-handed double helix to a higher energy alternative conformer such as Z-DNA [6]. Flipons act as switches to change the fate of transcripts, allowing compilation of different genetic programs from an initial set of RNAs [5–7].

There are many ways to form the Z-helix other than those powered by RNA polymerases. A helix that is one half left-handed and the other half right-handed forms when 2 single-stranded DNA circles are annealed [8], producing what was originally called form V DNA [9]. A similar outcome is possible when the ends of 2 complementary single-stranded RNAs or DNAs are constrained by protein complexes rather than by covalent bonds. Base pairing then creates sufficient topological stress within the bound segment to power a left-handed flip. Tangles, like those present in stress granules or arising during viral infection, appear when many ensnared RNA or DNA strands base pair with each other to form isolated topological domains [10]. The Z-RNA or Z-DNA formed during the process localizes Z-binding proteins to these regions [11].

### A biological role for left-handed conformations in dsRNA editing

**Fig 1:**
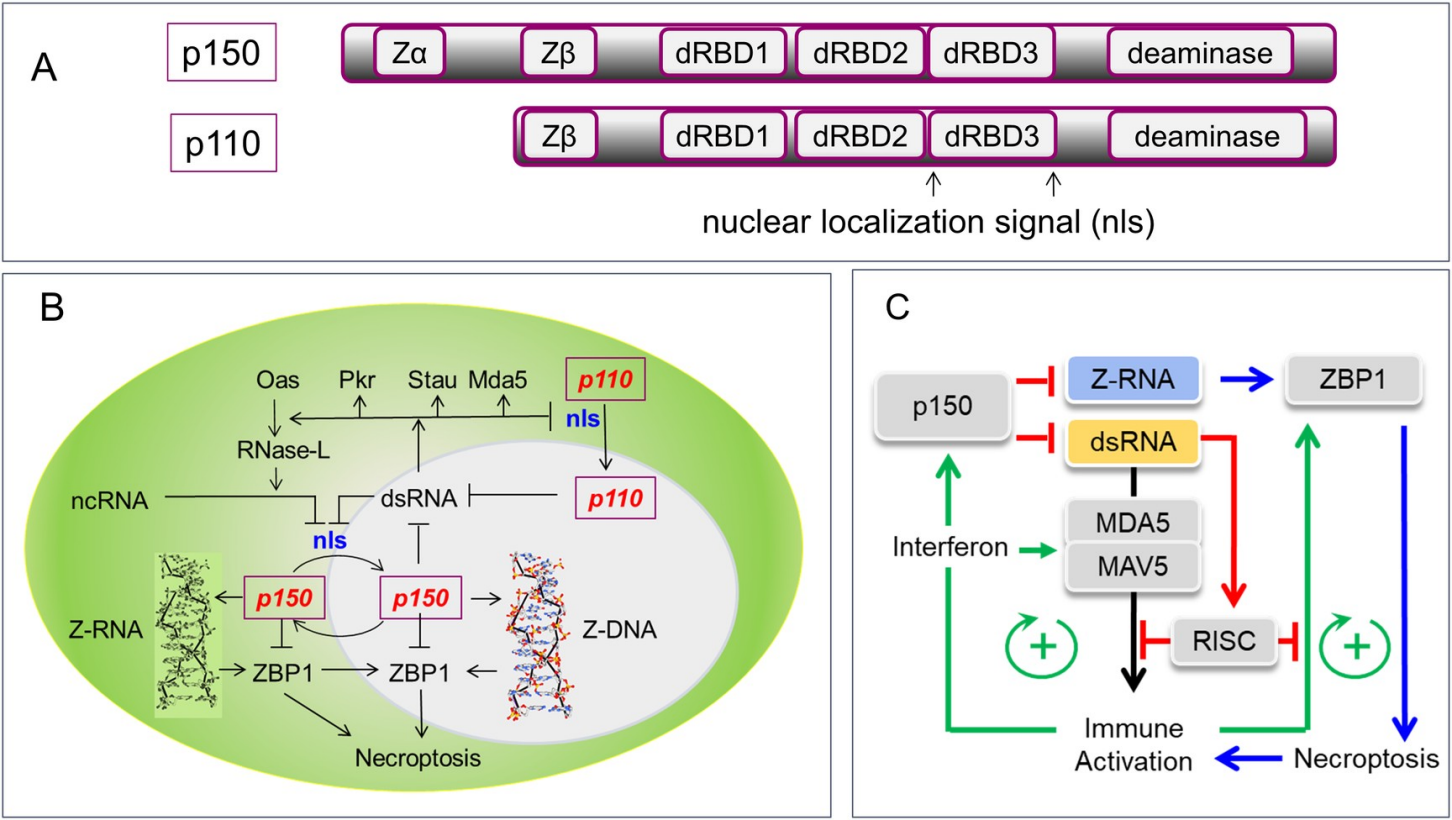
ADAR1 and Z-RNA. **(A)** Both p150 and p110 ADAR1 isoforms contain the same deaminase domain and dRBDs. Only ADAR1 p150 contains the Zα domain that binds both Z-DNA and Z-RNA. The function of the Zβ domain is not known. **(B)** A cell with the cytoplasm in green and the nucleus shaded gray. While p110 is mostly nuclear, p150 undergoes cytoplasmic shuffling. Both isoforms have a bipartite nls that spans the third dsRBD3 B. The nls is inhibited by dsRNA (indicated by a “T” shaped arrow) that can be generated by nuclear export or from cytoplasmic nuclease processing of misfolded or noncoding RNAs. Cytoplasmic dsRNA can also affect other pathways by targeting MDA5 (activates interferon responses), Stau (Staufen decay pathway) [40], Pkr (effects on RNA translation) [41], and Oas (2′-5′-oligoadenylate synthetase that activates RNase-L to cleave noncoding RNAs like tRNA, Y-RNA, and ribosomal RNA) [42]. **(C)** MDA5 bound to dsRNA activates the MAVS protein to greatly increase the expression of interferon, ADAR1 p150, ZBP1, and other genes. ADAR1 p150 destabilizes dsRNA and inhibits ZBP1 induction of necroptosis [43]. The RISC uses miRNAs to target self-sequences. Both the formation of miRNAs and availability of target sequences are modulated by helicases [99,100]. dRBD, double-stranded RNA binding domain; dsRBD3, dsRNA binding domain 3; MAVS, mitochondrial antiviral signaling; MDA5, melanoma differentiation–associated gene 5; miRNA, microRNA; nls, nuclear localization signal; Pkr, protein kinase R; RISC, RNA-induced silencing complex; ZBP1, Z-DNA binding protein 1.

An important step in understanding the biology of the left-handed Z-conformation was the identification of the Z-DNA specific, Zα binding domain in the dsRNA-specific editing enzyme ADAR1 (adenosine deaminase RNA specific, encoded by *Adar* in mice and ADAR in humans) [12]. This enzyme deaminates adenosine to form inosine, which is subsequently processed by the cellular machinery as guanosine [13]. The early focus was on the role this type of enzyme played in the recoding of mRNAs to produce new protein isoforms. The first example of a nonsynonymous codon change was the replacement of glutamine by arginine in the ion channel of the Gria2 receptor that changed its conductance properties [14]. Subsequent work attributed the recoding to a related enzyme ADAR2 (encoded by *Adarb1*), that lacked a Z-binding domain. When an edited allele was hardwired into the mouse genome, *Adarb1* knockouts had no phenotype, arguing that recoding of *Gria2* mRNA by this enzyme mostly compensated for a deleterious mutation [15]. The story with ADAR1 was more complicated. The gene encoded 2 isoforms: p150 that had the Z-DNA binding Zα domain and was induced by interferon and the constitutively expressed p110 (Fig 1A) [16]. They both share the same dsRNA binding and deaminase domains along with a Zβ domain, which, despite its sequence homology to Zα, is not known to bind Z-DNA [17]. The cellular location of the 2 isoforms varied. The p150 variant underwent nucleocytoplasmic shuffling, whereas p110 was mostly nuclear, with cytoplasmic accumulation occurring only when the bipartite nuclear localization signal (nls) spanning the third dsRNA binding domain was blocked by dsRNA (Fig 1A and 1B) [18] or phosphorylated by stress kinases [19].

### dsRNA editing substrates

As methods to analyze RNA transcriptomes evolved, it became apparent that transposable elements (TEs) were the major ADAR1 substrates, with few recoding events. The editing substrates most often arose from pairs of TE repeats orientated in opposite directions, enabling transcripts containing both to form dsRNA by base pairing with each other [20,21]. The TE were most often short interspersed nuclear repeats (SINES) from the Alu family. Alu elements constitute about 11% of the human genome and at one time were an existential threat, copying and pasting themselves indiscriminately into active genes [11]. Their modern day transcripts contain millions of RNA edits [22]. The question was “what was the function of all this editing?”

*Adar* knockout mice that lack both p150 and p110, or are homozygous for an editing null *Adar* variant, died by day 14 of development [23]. The embryos had a type I interferon signature due to activation of the dsRNA sensor MDA5 (encoded by *Ifih1*), a helicase that signals through the mitochondrial antiviral signaling (MAVS) protein (encoded by *Mavs*) pathway to induce interferon responses (Fig 1). Knockout of both *Adar* and *Ifih1* or *Mavs* provided partial rescue, with death a few days after birth [24,25]. Quite surprising was the complete rescue of an editing null *Adar* variant by the *Ifih1* knockout [24], demonstrating that just like ADAR2, recoding by ADAR1 played no role in development. The only reported phenotype was a delay in growth that was resolved within 12 weeks after birth. It became clear that the major function of ADAR1 was to inactivate endogenous dsRNAs that otherwise trigger the interferon pathway though MDA5 [26]. Knockout of the p150 isoform by deletion of *Adar* exon 1a, leaving expression of p110 intact, also produced an interferon signature and neonatal death [27]. However, unlike *Adar* null mice, most p150 deficient mice were completely rescued by *Mavs* deletion [25,28], providing the initial evidence that p150 and p110 isoforms have different roles, with only p150 down-regulating interferon responses.

### ADAR1 and human disease

In humans, genetic evidence that ADAR1 regulates innate immune responses was provided by studies in families with the Aicardi–Goutières Syndrome Type I Interferonopathy [29]. Here, deaminase domain loss of function variants produce autosomal recessive disease. A causal role for the ADAR1 Zα domain was confirmed by an extended analysis of the Zα P193A variant that is present in approximately 0.3% of non-Finnish Northern Europeans, suggesting positive selection in this population [30]. The identification of compound heterozygotes with one null allele allowed causal mapping of the P193A variant and another N173S variant directly to disease. The analysis exploited the haploid transcriptome present in these affected individuals [30]. Previous in vitro mutational scanning had identified both residues as essential to Z-DNA binding [31], findings supported by the Zα-Z-DNA crystal and NMR structures [32,33] and then by the structure of Zα bound to left-handed Z-RNA [34]. Separate studies also showed that many cancers are dependent on p150 to silence immune responses [35].

Subsequent experiments demonstrated that the mouse equivalent to the P193A variant recapitulated the human Zα mendelian phenotype [36]. Three other recently generated mouse models using different loss of function Zα residues variants also were associated with a type I interferon signature [37–39]. Taken together, the data confirmed the biological relevance of the Z-conformation and provided evidence for its role in the regulation of type I interferon responses. Questions then arose as to the nature of the p150-specific substrates, the triggers for Z-formation in vivo, and the pathways targeted.

### The importance of ADAR1 p150

Understanding the different roles for p150 and p110 in biology was not an easy task given the complexity of dsRNA-dependent pathways (Fig 1B and 1C). Besides MDA5, cytoplasmic dsRNA activates many other sensors, complicating the interpretation of results. The dsRNA targets include the Staufen RNA decay pathway [40], the dsRNA activated protein kinase R that regulates mRNA translation [41], and the 2′-5′-oligoadenylate synthetase enzymes that activate RNase-L to cleave noncoding RNAs like tRNA, Y-RNA, and ribosomal RNA to induce apoptosis [42]. ADAR1 p150 itself has a dual function. It not only destabilizes dsRNA but also modulates Z-RNA formation which is known to induce necroptosis by Z-DNA binding protein 1 (ZBP1) (Fig 1C) [43]. Both p150 and ZBP1 are induced by interferon. In contrast to ADAR p150, which down-regulates the interferon pathway, ZBP1 amplifies immune responses by producing immunogenic cell death (Fig 1C).

The analysis of functional differences between p110 and p150 initially started with expression of each isoform in cells that did not express ADAR1 protein. A common finding was that there was extensive overlap in the edits made by each isoform, with p150 edits occurring mostly in Alu elements, with around 50% occurring in 3′ untranslated regions (UTRs) in mice [44,45]. The further analysis of p150 biology in mice with Zα loss of function variants was confounded by the presence of p110 that edits many substrates in common with p150 as expected from their shared dsRNA and deaminase domains [37–39, 46]. In a technical tour de force, Kim and colleagues succeeded in creating a mouse that expressed only p150, but with no detectable p110 [47]. They did this by knocking out the p110 constitutive promoters and the associated exons 1b and 1c, while leaving the p150 exon 1a and the p150 interferon promoter intact. Previous attempts by these authors and other workers to remove p110 by mutating its initiation codon had failed due to the presence of a downstream methionine that yielded a truncated p110. With this new model, the authors were able to directly measure the editing of mRNAs targeted by p150 and its effects on interferon responses. The p150 only allele was alone sufficient to suppress the interferon signature in mice, even when expressed on a *Adarb1* null background. However, there was an increase in postnatal mortality. The early death phenotype could be reversed by creating a heterozygote with a p150 allele paired with an editing deficient p110 null allele, showing editing by p110 is not required for survival. In this cross, p150 expression was normal. The result confirmed that each ADAR1 isoform has a different role, with p110 playing no role in the interferon response.

The authors examined edited RNAs in the p150 only mouse. Overall, RNA editing is dramatically decreased, especially in the brain, occurring at 2% of wild-type levels. Most edits are in the 3′ UTR with very few in introns. Recoding of the known substrate AZIN1 is observed, most likely due to formation of a dsRNA substrate within the exon sequence [46]. Together, these findings suggest that in this model p150 editing is mostly cytoplasmic (Fig 1B). This makes sense as that is where activation of MDA5 by dsRNA normally occurs. In contrast, the models with loss of function Zα variants show an overall reduction of Alu-dependent intronic editing, suggesting that p150 can also act in the nucleus as a result of nucleocytoplasmic shuffling. One way to reconcile these findings is to postulate that p110 modulates the cellular distribution of p150 between nucleus and cytoplasm, by decreasing levels of cytoplasmic dsRNA that otherwise would block the nls and prevent nuclear uptake of p150 (Fig 1). This outcome could arise by the p110-dependent nuclear editing of dsRNA, causing its retention within the nucleus [48]. Alternatively, the p110 that accumulates in the cytoplasm during stress responses may act as a sponge for dsRNA [49]. Then p150 stays mostly cytoplasmic as nuclear uptake is inhibited by the free dsRNA that accumulates in the absence of p110. This explanation is supported by the finding that in the Zα loss of function mice, which have normal p110 levels, the nuclear and cytoplasmic levels of p150 are the same as wild type [37].

### Z-RNA formation by the helicase MDA5

**Fig 2:**
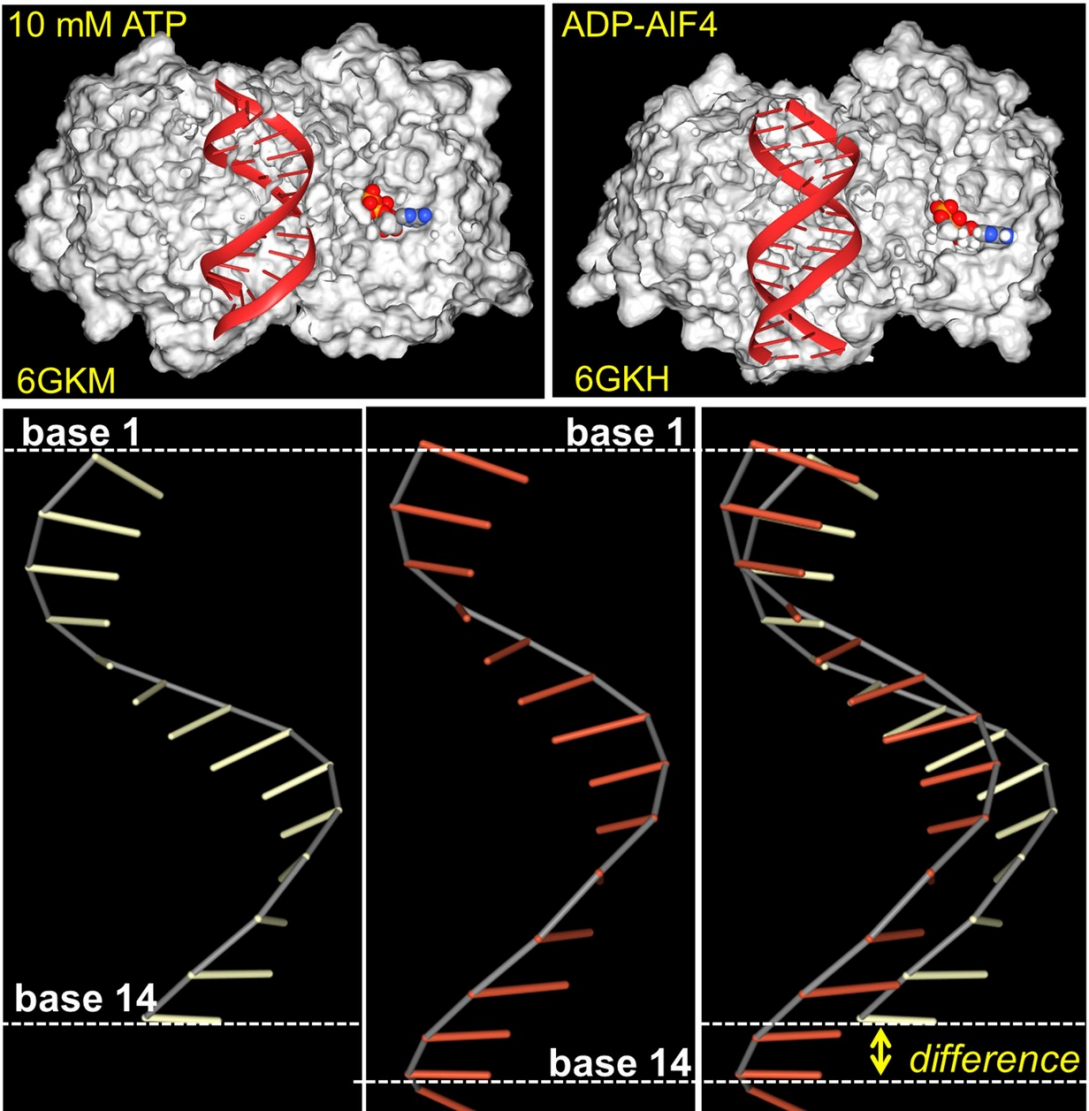
The dsRNA helix is shortened in ATP-bound MDA5 filaments. The upper figures show MDA5 bound to dsRNA and to either ATP (upper and lower left) or to the ATP transition state analog adenosine diphosphate-tetrafluoroaluminate (ADP-AIF_4_) (upper right and lower middle). A cross section of the surface representation of the protein allows visualization of the bound dsRNA and the nucleotide ligand (to the right of the dsRNA in the upper images). The lower figures show only 1 strand of the RNA double-helix (dsRNA) from the cryo-EM structures described in PDB records 6GKM and 6GKH to emphasize the difference in the relative position of the RNA base 14 in the 2 complexes due to differences in how the filament twists [49]. The compact MDA5 complex in the ATP bound form has 5 MDA5 subunits per step, corresponding to a twist of approximately 72° for each. The ADP-AIF_4_ complex has 4 subunits per step, corresponding to a twist approximately 90° for each [49]. The extra twist of the ATP filament is associated with an increased inclination of the bases [83]. It creates torsion of the adjacent dsRNA that can be relieved by unwinding the dsRNA into single-stranded RNAs [49]. Under the model described here, the strain is released by flipping the right-handed A-form dsRNA (24.6 Å) to the longer left-handed Z-RNA conformation to (45.6 Å), triggering ATP hydrolysis as MDA5 releases. ATP, adenosine triphosphate; dsRNA, double-stranded RNA; MDA5, melanoma differentiation–associated gene 5; PDB, Protein Data Bank (https://www.rcsb.org/).

**Fig 3:**
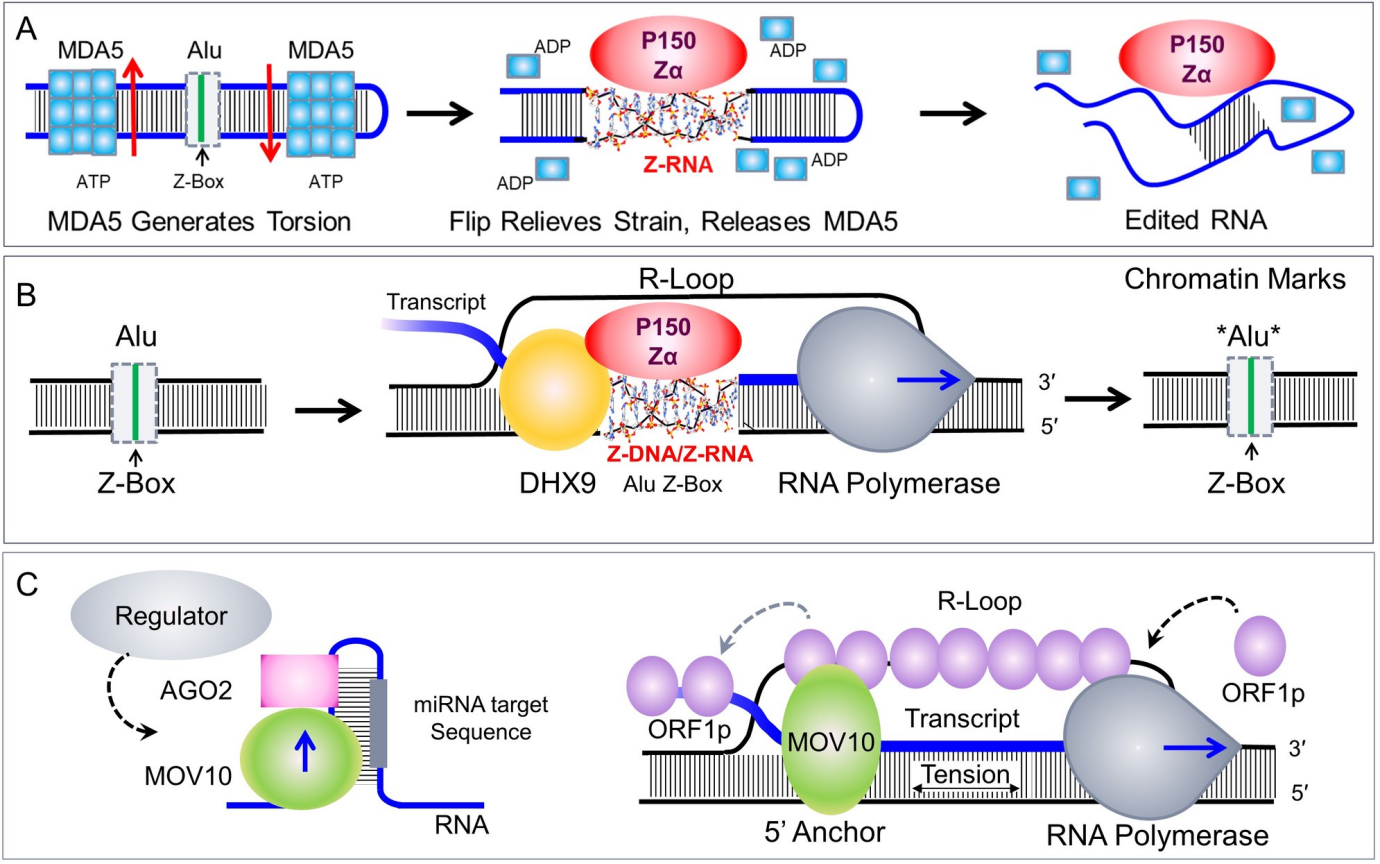
Different roles for helicases in self-recognition, with RNA strands drawn in blue and DNA strands in black. **(A)** It is proposed that MDA5 induces Z-RNA formation in dsRNA by twisting and shortening the dsRNA. The strain induced promotes the flip to the longer Z-RNA helix, acting like a pull-switch to stop MDA5 filament formation. Alu retroelements contain a highly conserved Z-Box with sequences that favor Z-formation. Once Z-RNA is engaged by its Zα domain, ADAR1 deaminates adenosine to form inosine, destabilizing the dsRNA and preventing MDA5 filament formation. Both outcomes prevent the activation of interferon responses. **(B)** It is proposed that another helicase, DHX9, anchors R-loops when splicing is inhibited, localizing ADAR1 p150 to Alu Z-boxes in underwound regions of the DRH where Z-formation occurs, altering the transcript produced. Epigenetic marks on Alu chromatin following transcription are indicated by an asterisk—they threshold future responses. **(C)** The MOV10 normally unwinds dsRNA to allow the access of miRNAs and AGO2, a component of the RISC to their target sequences. This mechanism is one where specific self-sequences recognition enables suppression of immune responses in the normal state [100]. Alternatively, proteins, such as and FMRP [99] stabilize the MOV10 complex with its substrate and protects transcripts from suppression by RISC. It is proposed that a similar type of interaction with the LINE LI ORF1p locks MOV10 in place, anchoring the 5′ end of the transcript. The DRH formed are targets for RNaseH2. As described in the text, segments within the DRH may flip to the Z-conformation, causing ZBP1 activated necroptosis. With the helicase fixed in place, the movement of RNA polymerase tensions the element, powering the flip. AGO2, Argonaute2; DHX9, DExH-box helicase 9; DRH, DNA:RNA hybrid; dsRNA, double-stranded RNA; FMRP, fragile X mental retardation protein; MDA5, melanoma differentiation–associated gene 5; miRNA, microRNA; MOV10, Mov10 RISC complex RNA helicase; ORF1p, open reading frame 1 protein; RISC, RNA-induced silencing complex.

Collectively, the findings show that p150 is a negative regulator of type I interferon responses and that this outcome is dependent on a functional Zα domain. They demonstrate that one role for Z-RNA is to protect against interferon induction by self-RNAs (Fig 1C). Here, MDA5 acts as a dsRNA sensor that assembles on long dsRNAs to provide a scaffold for the activation of downstream effectors. Previously, it was proposed that MDA5 proofreads dsRNA and identifies those that signal danger by using mechanical stress [49]. In this model, ATP increases the MDA5 filament twist, generating torsion that unwinds any dsRNA substrates too short to support filament elongation and MAVS activation (Figs 2 and 3A). Unwinding is associated with ATP hydrolysis. However, dissociation into single strands is not possible when both ends of a dsRNA are fixed. I propose that another way to relieve the MDA5 induced stress is by formation of Z-RNA. The Z-helix is much longer than the A-helix, with a helical pitch of 45.6 Å compared to that of 24.6 Å for A-form dsRNA. The flip to Z lengthens the dsRNA substrate, relaxing the tension generated by MDA5, triggering dissociation and ATP hydrolysis. The twist mechanism is analogous to the way an electrical pull switch works. The chemical and mechanical energy expended is captured by the conversion of A-RNA to its higher energy Z-RNA conformer. Fueling the formation of alternative nucleic acid structures like Z-RNA constitutes a novel role for DEAD box helicases like MDA5.

Alu inverted repeats (AIRs) are the most common sequences in the human genome that form dsRNA. They contain a highly conserved sequence block, referred to as a Z-Box, that is prone to Z-formation [11]. That these AIR sequences are really Z-RNA forming flipons was recently confirmed in NMR studies [50], with noncanonical base pairs lowering the energetic cost of formation. The flip allows AIRs to engage the Zα domain of p150, explaining their high levels of editing and the destabilization of the dsRNA they form. A number of factors determine the extent of editing by p150. The propensity to form Z-RNA depends upon the Z-Box base composition and the modifications present [5,11]. The level of editing also depends on the number of AIRs in a transcript, varying with their length, splice history, and the site of polyadenylation. The impact is greatest for 3′ UTRs, affecting the stability and translation of a large number of mRNAs [40,51]. In addition, RNA base modifications outside the Z-Box play an important role. For example, N^6^-methyl adenosine marks restrict MDA5 filament formation [52], potentially leaving some dsRNA segments free to form Z-RNA. This modification and others like ribose 2′-O-methylation create a molecular signature to identify self, one many viruses have usurped in one form or another [53].

### Alternative models of self-recognition by MDA5

Z-RNA formation by AIRs provides a way to distinguish self-RNAs from those of pathogens. Their Z-Box is highly conserved, supporting the proposed role in modulating immune responses against self. The data that p150 is key to negatively regulating interferon responses and that loss of function variants the Zα domain are associated with type I interferonopathies provide strong evidence for this mechanism. The model incorporates many elements of previous proposals on how MDA5 discriminates nonself from self that are based on the localization, structure, and availability of dsRNA [54–58]. None of these earlier models completely fit with the data. One idea was that viruses produce dsRNA much longer than the host. Over time, those hosts that survive increase their advantage by eliminating long dsRNA from their genomes by purifying selection, making those formed by attacking viruses easier to sense [59]. While this may happen, there still remains a substantial reservoir of long dsRNA encoded in the human genome. An analysis of the dsRNAome in humans reveals 3,438 dsRNA editing enriched regions with an average length of 845 base pairs [60]. Over 80% of these regions are in introns [61]. In mice, only 382 regions are reported [60], with over 80% in the 3′ UTR [61]. Estimating the amount of long dsRNA produced from the genome is further complicated by the variations in the length and sequences of RNA that arise from noncanonical RNA processing. Circular RNAs and the occurrence of intron retention can produce long-lived RNAs that incorporate repeat elements capable of pairing to form dsRNA. One class of circular RNA derives from back splicing due to self-complementary sequences within an intron [62]. It is diminished by ADAR1, suggesting the importance of Alu elements in this process, as well as by DHX9, a helicase with a preference for Alu repeats [63]. Intron retention also can occur in linear sequences. It acts to increase protein isoform diversity [64] and enables rapid responses by delaying splicing until the protein product is required [65]. As discussed below, introns also can be processed to produce cytoplasmic dsRNA [66]. The importance of such processes in currently an area of active investigation. Only recently have the technical challenge to fully documenting the extent and consequences of these noncanonical RNA processing pathways been solved. How they alter the dsRNAome and MDA5 activation is currently unresolved [64,67].

Doubts have been expressed about whether it is the length of the dsRNA or some other feature that is important. Some higher ordered structure in long dsRNAs appears necessary to activate MDA5 [68]. MDA5 shows substrate specificity, raising questions whether the length of the dsRNA alone is sufficient to explain its activation of MDA5. Although the identification of MDA5 substrates has been challenging [57], a recently developed in vitro MDA5 protection assay [26] reveals that AIR sequences are enriched in the RNAs bound by MDA5 from untreated human cell lysates. Around 35% of the AIRS are encoded in 3′ UTRs [66]. In unperturbed cells, transcription of most AIRs is suppressed by methylation, but their expression can be induced by the DNA methyltransferase inhibitor (DNMTi) 5-AZA-CdR. After DNMTi treatment, around 90% of AIRs bound by MDA5 are transcribed from introns. They undergo polyadenylation and accumulate in the cytoplasm. DNMTi treatment also stimulates ADAR1 p150 transcription and increases A-to-I editing of transcripts [66]. In these experiments, depletion of ADAR1 by combinations of short hairpin RNAs increases MDA5 activation. The findings are consistent with earlier results showing that ADAR1 p150 acts subsequently to the initial activation of the MDA5 pathway, with the time course of MDA5 driven responses extending over days [26]. MDA5 expression is interferon inducible, a key factor in the amplification of the initial response (Fig 1C). The additional filaments formed on host-derived AIRs by newly synthesized MDA5 then create a positive feedback loop that further augments outcomes. This loop is inhibited by ADAR1 in wild-type cells but not in cells with loss of function ADAR1 variants. Collectively, the findings are consistent with the model presented here where MDA5 induced formation of Z-RNA localizes p150 to AIRs, inhibiting further activation of an interferon response driven solely by self-transcripts. Other inhibitory mechanisms that act directly on MDA5, such as protein phosphorylation, maintain quiescence in resting cells until the equilibrium is perturbed [57]. This proposed role for p150 is further evidenced by the association between an infectious or febrile episode and the onset of Zα-dependent mendelian disease [69]. Once the interferon response is initiated, the ADAR1 loss of function variants are unable to fully curtail the amplification loop [30]. The negative regulatory circuit involving Z-RNA formation is a simple but elegant evolutionary outcome where Alu elements, once the invader, are now co-opted to protect the host.

### Viral counter responses

So, can viruses and other invaders exploit this mechanism to masquerade as self or to promote their own replication? They can and certainly do. Many viruses produce Z-binding proteins [70] that can modulate Z-dependent host responses such as the ZBP1 dependent necroptosis of host cells [43,71], while ADAR1 p150-dependent editing of their genomes increases variability [51] and increases infectivity by decreasing packaging of defective genomes [72]. The role of p150 in other infections is less certain, although ADAR1 impacts the virulence of many viruses [73]. An example is provided by the hepatitis delta virus (HDV), where envelopment requires production of the large HDV antigen. This event depends upon editing of HDV by ADAR1, and requires interferon, suggesting the involvement of p150 [74,75]. Why this requirement exists is not known, but one possibility, so far not investigated, is that there is a propensity of HDV to form Z-RNA during viral packaging leading to ZBP1 activation. The potential mechanism for Z-formation is similar to those described above and is due to dsRNA created by the extensive base pairing within many regions of the single-stranded circular viral genome or those that involve different copies and create dsRNA tangles. Even though the genome is only 1,700 bases long, topological stress sufficient to form Z-RNA in unbound dsRNA segments or in viral dsRNA tangles may arise during the coating of viral dsRNA with capsid protein. This virus then is vulnerable to host Z-RNA dependent defenses, with p150 engagement offsetting the risk of ZBP1 induced necroptosis. Other viruses also have very specific packaging requirements to ensure that they maintain infectivity. In the case of influenza with its linear negative-sense, single-stranded RNA genome, segments minimally base-pair to ensure that they are all packaged into the virion [76], while with retroviruses, packaging requires formation of a dsRNA dimer from 2 single-stranded, positive-sense, linear RNA molecules [77,78]. The choreography of wrapping these small dsRNA regions in protein could also generate topological stress capable of inducing Z-RNA formation and ZBP1 activation, especially in the RNA tangles formed from defective genomic copies. Here again, competition by ADAR1 would be protective. Z-RNA formation by viruses in these cases occurs independently of MDA5.

### Not all helicases are created equal

It is reasonable to ask whether MDA5 is unique in promoting self-recognition or is this a more general property of helicases. There are 117 different helicases annotated in the human genome [79] that differ in the way they twist, stretch, pull, or flip their substrates; in whether or not their optimal substrate is DNA, RNA, or neither; or whether they are 3′ or 5′ processive, or not at all; or whether they require substrates with free ends or not [80,81]. Here, the focus is on dsRNA substrates with both ends fixed, either by protein complexes or because of extensive cross hybridization with other RNAs or because they involve covalently, closed circular single-stranded RNAs that base pair with each other. Such structures are able to trap within their bounds the energy needed to power the formation of alternative nucleic acid conformations like Z-RNA.

Helicases can regulate Z-formation in different ways. Like MDA5, one action of helicases is to change the twist of their double-stranded substrate. The term twist measures the rotation of one base relative to an adjacent one. A change in twist affects dsRNA and dsDNA differently. While twisting decreases the length of a dsRNA helix (Fig 2), dsDNA helices rather surprisingly elongate [82,83]. To achieve the same outcome, it is necessary to twist RNA and DNA in opposite directions. As we discussed for MDA5 and dsRNA, flipping the right-handed A-helix to the left Z-RNA is one way to relieve the stress generated. Formation of other non-A structures or filaments with a stretched backbone are also possible outcomes. For example, the filaments formed by Rad51 and RecA recombinases increase the helical rise by expanding the step size from 3.3 Å in B-DNA to 5.1 Å [84,85]. Other helicases instead of twisting, pull. The mechanical force is delivered analogously to the way a winch works when it winds in rope. Some helicases employ such a strategy to unravel quadruplex structures [86]. Surprisingly, the tension generated can induce alternative conformations like Z-DNA when the duplex is tethered at the other end. This principle has been elegantly demonstrated with single-molecule approaches using magnetic tweezers to tug on DNA [87,88]. Biologically, another mechanism exists by which helicases can generate tension. In this case, a helicase provides an anchor for the 5′ end of a transcript while a transcribing RNA polymerase pulls at its 3′ end (Fig 3C). The tension produced then depends on the relative motion of the polymerase and the helicase. Experimentally, the mechanical force a polymerase generates when pulling on a transcript with a fixed end is sufficient to flip an alternative conformation [89,90]. In the present context, the combination of a helicase with an RNA polymerase presents a powerful mechanism for regulating the local topology of a double-stranded helix. It is a different strategy from the twisting action used by MDA5 to generate Z-RNA, but one still capable of modulating immune and other responses, as discussed below.

### DHX9-type helicases

Currently, there is insufficient information to say for sure whether other helicases induce Z-formation, but given the diversity of helicases and the available data, it is likely. One characteristic of the Superfamily 2 (SF2) helicases, including MDA5, is that they do not slide along a duplex. Instead, they clamp onto their substrate, initiate local duplex unwinding, hydrolyze ATP as they release, then reattach [79,91]. One candidate to modulate Z-formation is DHX9 that, like MDA5, preferentially binds Alu DNA elements. DHX9 also physically interacts with ADAR1 p150 (Fig 3B) [63,92]. When splicing is inhibited, either by splice-factor knockdown or with small molecules, DHX9 forms DNA:RNA hybrids [93]. It enhances canonical splicing and mRNA translation while decreasing formation of the noncanonical circular RNAs as described above [63]. What causes these outcomes? One possibility is that Z-formation by the DNA:RNA hybrid (DRH) and subsequent editing by p150 alters how transcripts are processed [7]. In this scenario, DHX9 tethers the 5′ end of the hybrid, delaying release of the transcript until the appropriate complexes assemble to process the nascent RNA (Fig 3B). The cycles of bind and release would cause the elongation rate to vary and the leading RNA polymerase to jitter. Both effects are experimentally detectable. Such a mechanism likely ensures that transcripts are edited prior to splicing and that sufficient heterogeneous nuclear ribonucleoproteins are loaded onto the RNA for subsequent processing. Colocalizing DHX9 with proteins like p150 also helps target Z-dependent edits to a specific subset of self-transcripts to modulate immune and other responses. At times of cellular stress, the targeting of p150 provides a way to prevent the accumulation of DRH that might otherwise be deleterious. Although DHX9 shares with MDA5 a preference for Alu elements and both involve p150 in their biology, the outcomes differ.

Besides DHX9, other DEAD box RNA helicases like DDX3, DHX29, DHX36, and DDX60 regulate type I interferon production during viral infection [94]. They form nucleofilaments that could potentially modulate flipon conformation, with outcomes varying by context and cell type. Both DHX36 [86].and DHX5 [95] bind G4-quadruplexes. DHX5 impacts c-MYC gene expression and alters splicing during epithelial-to-mesenchymal transdifferentiation [96].Their effects on interferon responses differ from those of MDA5 and in some cases are virus specific [97]. The lack of functional redundancy in this family is consistent with its rapid evolution in response to emergent threats.

### MOV10 like helicases

MOV10 is a 5′ to 3′ processive helicase, belonging to a different helicase family (SF1) than DHX9 and MDA5 [98]. Expression is enhanced by interferon, providing protection against RNA viruses in a MAVS independent manner. MOV10 has many functions related to both transcription and translation. It associates with the RNA-induced silencing complex (RISC), either increasing microRNA (miRNA) efficacy by exposing targets buried in dsRNA, or by anchoring complexes, like the one with fragile X mental retardation protein (FMRP) that prevent target recognition from occurring (Fig 3C) [99]. In the case of interferon responses, RNA binding, but not helicase activity, is required. By unwinding dsRNA, MOV10 enables the recognition of self-sequences by miRNAs, resulting in the suppression by RISC of those mRNAs that otherwise might initiate and amplify an immune response (Fig 1C) [100]. By forming complexes that leave miRNA targets buried in dsRNA, MOV10 is able to generate interferon responses as RISC can no longer suppress them. This mode of immune regulation is also based on self-recognition, but one that is sequence specific and different from the structure-based response involving MDA5.

MOV10 also inhibits LINE L1 retrotransposition. Knockdown of MOV10 promotes in vivo L1 mobilization, which involves formation of DRH [101]. The DRH potentially play a role in activating ZBP1, as nucleotide reverse transcriptase inhibitors, thought to inhibit the LINE-L1 retrotransposase [102], decrease ZBP1-dependent necroptosis in a mouse model of skin inflammation in which the expression of endogenous retroelements, including LINE-L1s, is elevated [103]. Ribonuclease RNaseH2, which cleaves DRH, also restricts L1 transposition. That outcome depends on MOV10 helicase activity [101] and its interaction with the LINE-L1 open reading frame 1 protein (ORF1p) [104], suggesting that the host is using MOV10 to stabilize the LINE-L1 DRHs that RNaseH2 and ZBP1 target. One possibility is that the interaction of MOV10 with ORF1p anchors the 5′ end of the LINE-L1 transcript. MOV10 then promotes Z-formation powered by the transcribing polymerase, rather than releasing the nascent RNA from the hybrid (Fig 3C).

### What next?

Just as phage screens advance the understanding of codon biology, we are now in the position to develop analogous genetic approaches to understand how flipons operate. The MDA5 system represents a minimal flipon system with only 3 major components: MDA5, the Zα domain, and the flipon. Each partner can be manipulated to explore the role of environmental perturbations and base modifications in outcomes. The system is amenable to genetic screens for discovering response modifiers. Mouse models with either gain or loss of functions MDA5 and Zα variants are available for in vivo studies to better understand the role of Alu Z-flipons in disease [105]. The LINE L1 system likely presents another opportunity for testing the impact of flipons on retrotransposition. L1 retrotransposition depends on the formation of DRH. That the hybrids flip to Z is supported by the suppression of LINE L1 ZBP1 activation by reverse transcriptase inhibitors [103]. The 2 key host regulators in this system, MOV10 and RNaseH2, potentially also modulate Z-formation [101]. There are high-throughput assays available to perform unbiased screens to examine the role of flipons in this system [101]. Questions concerning how Z-formation impact alternative splicing [106] and polyadenylation [107] can also use L1 transcripts as a model system.

## Summary

The induction of Z-RNA formation in AIR elements by MDA5 offers a general approach for the recognition of self-transcripts and for the assembly of complexes essential to ADAR1 p150-dependent RNA editing, stability, splicing, and translation of RNAs. Other helicases also have the potential to moderate formation of alternative structures like Z-RNA, Z-DNA, and quadruplexes with different outcomes than those produced by MDA5. The repeat sequences involved and the events leading to the flip may differ [7]. For helicases like MOV10, the recognition of self-transcripts likely depends on the unmasking of specific host sequences by unwinding dsRNA structures that hide them. Targeting of these transcripts by noncoding RNAs then prevents induction of an interferon response. Collectively, the diverse assortment of helicases tempers immune attacks against self-transcripts while enabling them against viruses. They protect the host while targeting threats. In each case, helicases switch the outcome by modulating DNA and RNA conformation, not by recognizing specific nucleotide sequences [5]. This class of enzymes is likely to play a much broader role than currently appreciated in dynamically shaping host responses to a constantly changing environment [5]. The alternative nucleic acid conformations they modulate, based mostly on low complexity repeats [7], likely increase transcript diversity by changing the parsing of genomic information, creating phenotypic variability for natural selection to act upon. The co-option of Alu retrotransposons to mark self is a remarkable example of how genomes evolve existential threats into something essential for their survival. In the case of MDA5, Alu Z-flipons switch innate immune responses off.

**Key Learning Points**

- The p150 isoform of the double-stranded RNA (dsRNA) editing enzyme ADAR1 is a negative regulator of the type I interferon response induced by the dsRNA sensor melanoma differentiation–associated gene 5 (MDA5).
- MDA5 forms a filament on dsRNA, assembling in a patchwise fashion. The helicase twists and shortens the dsRNA segment as it binds. This process induces topological stress in the unbound dsRNA between the MDA5 patches.
- The stress can be relieved by flipping A-form dsRNA, with a helical length of 24.6 Å, to the longer Z-RNA helix that has a helical length of 45.6 Å. The transition triggers MDA5 release and ATP hydrolysis.
- The Z-RNA that is formed localizes ADAR1 p150 to the dsRNA through its Zα domain.
- The dsRNA bound by MDA5 and targeted by ADAR p150 is often formed by base pairing of inverted repeat elements that fold back on themselves.
- The inverted repeats also contain sequences, called flipons, that form Z-RNA under physiological conditions. By forming Z-RNA, the repeat elements dissociate MDA5 filaments and localize editing by ADAR p150 to host transcripts, thus preventing the activation of interferon responses targeting self dsRNAs. These repeat elements, mostly spread through retrotransposition, once the invader, are now co-opted to protect the host.

**Top Five Papers**

1. Herbert A, Lowenhaupt K, Spitzner J, Rich A. Chicken double-stranded RNA adenosine deaminase has apparent specificity for Z-DNA. Proc Natl Acad Sci U S A. 1995;92(16):7550–4. Epub 1995/08/01. doi: 10.1073/pnas.92.16.7550. PubMed PMID: 7638229; PubMed Central PMCID: PMC41377.
2. Athanasiadis A, Rich A, Maas S. Widespread A-to-I RNA editing of Alu-containing mRNAs in the human transcriptome. PLoS Biol. 2004;2(12):e391. Epub 2004/11/10. doi: 10.1371/journal.pbio.0020391. PubMed PMID: 15534692; PubMed Central PMCID: PMC526178.
3. Ward SV, George CX, Welch MJ, Liou LY, Hahm B, Lewicki H, et al. RNA editing enzyme adenosine deaminase is a restriction factor for controlling measles virus replication that also is required for embryogenesis. Proc Natl Acad Sci U S A. 2011;108(1):331–6. Epub 2010/12/22. doi: 10.1073/pnas.1017241108. PubMed PMID: 21173229; PubMed Central PMCID: PMC3017198.
4. Liddicoat BJ, Piskol R, Chalk AM, Ramaswami G, Higuchi M, Hartner JC, et al. RNA editing by ADAR1 prevents MDA5 sensing of endogenous dsRNA as nonself. Science. 2015;349(6252):1115–20. Epub 2015/08/15. doi: 10.1126/science.aac7049. PubMed PMID: 26275108; PubMed Central PMCID: PMC5444807.
5. Herbert A. Mendelian Disease caused by variants affecting recognition of Z-DNA and Z-RNA by the Zα domain of the double-stranded RNA Editing Enzyme ADAR. European Journal of Human Genetics. 2019. doi: 10.1038/s41431-019-0458-6.
